# Developing the quality evaluation index system of digital textbooks for physical education and health

**DOI:** 10.3389/fpsyg.2026.1825248

**Published:** 2026-09-07

**Authors:** Yixiao Zhang, Zhihua Yin, Qiying Zhong, Qingyuan Zhou, Mingzhu Sun, Changsi Zhang

**Affiliations:** 1 College of Physical Education and Health, East China Normal University, Shanghai, China; 2 Department of Physical Education Teaching, Shanghai University of Engineering Science, Shanghai, China; 3 College of Physical Education and Sports, Beijing Normal University, Beijing, China

**Keywords:** digital teaching materials for physical education, digital transformation of education, evaluation indicators, physical education curriculum, quality of development

## Abstract

**Background:**

Digital textbooks have evolved beyond mere digital replicas of printed versions. They have transformed into a new type of teaching resource that integrates multimedia elements, intelligent interaction, dynamic learning analytics, and data-driven support. The research and application of digital textbooks for physical education have become a crucial component of advancing the digital transformation of physical education. This study focuses on the quality of the development of digital textbooks for physical education, aiming to construct a scientific and comprehensive evaluation index system that provides theoretical basis and practical guidance for the development, application, and evaluation of digital textbooks, and to contribute to the modernization of education.

**Methods:**

The study first reviewed relevant achievements in the evaluation of digital textbooks and physical education and health in China and abroad through literature review, clarifying the connotation, elements, and major problems of digital textbooks for physical education. Then, it applied the Delphi method and the analytic hierarchy process, and the index selection, structure revision, and weight determination were completed through multiple rounds of expert consultation. Finally, mathematical statistics were used to analyze reliability, validity, and consistency on the expert data to ensure the objectiveness and reliability of the constructed evaluation system.

**Results:**

This study constructed a quality evaluation index system for digital textbooks for physical education in primary and secondary schools, comprising 4 primary indicators, 16 secondary indicators, and 100 tertiary indicators. The system encompasses key dimensions—educational orientation, content structure, teaching applicability, and compilation standards—thereby reflecting the textbooks’ value alignment, scientific rigor, practical applicability, and standardization. The weight analysis revealed that “Content structure” and “Teaching applicability” carry the highest weights (both 0.351), followed by “Compilation standards” (0. 189), while “Educational orientation” has a relatively lower weight (0. 109). Among the secondary indicators, “Value Orientation” “Content Presentation Standards” and “Structural System” are ranked first within their respective dimensions.

**Conclusion:**

The quality evaluation index system for digital physical education textbooks developed in this study is both scientific and operable. It provides systematic theoretical support and a practical basis for the research, review, and continuous improvement of digital textbooks. In response to current problems—such as outdated content, monotonous presentation formats, insufficient application in teaching, and weak feedback mechanisms—this study proposes strengthening textbooks by optimizing content, innovating in presentation, and improving teaching support and feedback. These measures are intended to enhance the timeliness, interactivity, teaching adaptability, and continuous improvement capabilities of the textbooks. In the future, the indicator system should be dynamically adjusted alongside changes in education policies and technological evolution. This will continuously improve the quality and innovation level of digital textbooks, allowing them to better serve teaching practice and promote students’ all-round development.

## 1 Introduction

As digital transformation of global education advances, digital textbooks have become a pivotal component of curriculum reform across basic and higher education. International organizations such as UNESCO and OECD highlight the need to improve the accessibility, interactivity and adaptability of digital educational resources.

Moreover, recently the Chinese government has called for the digital transformation of education. The “Compulsory Education Curriculum Scheme (2022 Edition)” and the “Compulsory Education Physical Education and Health Curriculum Standards (2022 Edition) “explicitly propose to elevate the quality of digital textbook development and the deep integration of curriculum content with digital technology (AlKasasbeh and Amawi, 2024). International research offers digital textbooks diverse perspectives. For example, Elliott and Kim (2025) explored the potential of AI digital textbooks to overcome barriers in cross-cultural education. Morita et al. (2025) provided a technical paradigm for embedding intelligent assessment and feedback mechanisms by visualizing data within digital textbooks to evaluate learner comprehension. Wijayanti et al. (2025) demonstrated the positive impact of digital textbooks on narrative writing skills, which also provided a methodological reference for empirical research on digital textbooks for physical education, particularly concerning motor skills acquisition. International reviews further show that physical education textbooks and learning materials should be evaluated across curriculum contexts (Rodríguez Rodríguez et al., 2022).

As a discipline focusing on physical practice and all-round student development, physical education faces unique challenges in digital transformation. Current digital resources cannot fully match its practical teaching modes. Physical education is a discipline focused on physical activity and student competency development. Its digital transformation effectively narrows the urban–rural educational gap. Digital textbooks for physical education enable the systematic organization and presentation of knowledge, skills, methodologies, and contextual elements through digital means, constituting an essential component of physical education curriculum materials.

H’mida et al. (2020) and Jastrow et al. (2022) pointed out that digital teaching materials can overcome the static limitations of traditional printed textbooks and present complex sports skills in an intuitive and dynamic manner through multimedia formats such as videos and animations, effectively resolving the pain points that teachers struggle to demonstrate and students grapple with understanding in teaching.

Despite rapid advancement of digital textbooks for physical education in recent years, many challenges persist. For example, some resources overemphasize visual presentation while ignoring disciplinary logic and teaching goals. Existing quality assessments for digital textbooks mainly focus on general subjects such as language and mathematics. These frameworks pay excessive attention to technical performance and user experience, but fail to match the practical, dynamic and interactive attributes of physical education. They cannot evaluate core teaching functions including motor skill demonstration and on-site learning guidance, resulting in the absence of targeted evaluation standards for physical education digital textbooks. Gu et al. (2015), for example, found that many current digital textbooks are merely digitized versions of printed materials—merely transferring content to digital devices without fully leveraging digital technology to create deeply interactive and contextualized learning experiences. Lei (2024) further noted that digital textbooks are either built but not used, or used but not properly integrated into actual teaching, showing insufficient connection to classroom instruction, extracurricular practice, and health assessment. At the same time, domestic and international research] on the evaluation of digital textbooks are mostly limited to core subjects such as language, mathematics, and science, lacking a quality evaluation system for digital textbooks for physical education. Wang and Jin (2024) further highlighted the value of digital textbooks in promoting educational equality and achieving personalized learning. Digital resources can transcend time and space, providing students with customized learning paths and immediate feedback. These studies sufficed for the necessity and legitimacy of digital textbook, serving as a fundamental starting point for constructing quality evaluation indicators. Furthermore, Brnic et al. (2024) compared the learning effects of digital and printed materials on students of different genders, and Im (2024) conducted a longitudinal study on the impact of digital textbooks on students’ emotional and social competence. Both called for student-centered evaluation perspective, inspiring research to focus on the actual impact of digital textbooks on students’ learning motivation, skill mastery, and comprehensive literacy. Inclusive representation should also be considered, as disability representation has been examined in physical education textbooks (González-Palomares and Rey-Cao, 2022).

Therefore, it is urgent to construct a quality evaluation indicator system for digital physical education textbooks. Different from previous general digital textbook evaluation systems, this study fully targets the practical characteristics of physical education, focuses on skill teaching and embodied learning, and builds a three-tier index framework covering educational orientation, content structure, teaching applicability and compilation standards. It not only supplements the disciplinary evaluation standards in this field, but also provides operable assessment tools for practical application. This system should not only cover key dimensions such as the scientific nature of content, the logical structure, the technological adaptability, and the learning facilitation, but also fully reflect the subject attributes and goals of the physical education curriculum, highlighting how well the digital textbooks contribute to the development of students’ core physical education competencies, so as to ensure that digital textbooks truly serve the core values and practical requirements of the physical education and health curriculum. This study adopts a hybrid research method, combining literature review and expert interviews to identify the core elements of the quality of digital textbooks for physical education and construct a corresponding evaluation index system, aiming to provide a systematic reference for textbook development, policy formulation, and teaching practice.

## 2 Materials and methods

This study developed evaluation indicators for the quality of digital textbooks for physical education in three phases. The first phase encompassed defining digital teaching materials through literature retrieval. The second phase used the Delphi method to solicit expert opinions to determine the evaluation indicators. The third stage involved experts determining the weights of the evaluation indicators.

### 2.1 First stage: clear definition and initial indicators

#### 2.1.1 Defining digital textbooks for physical education

This study takes digital textbooks for physical education as its core research object. A clear and sound definition of digital textbooks for physical education is crucial, contributing to identifying the scope of research and supporting the validity of the indicator system and measurement results. Currently, there is no clear consensus in academia on “digital textbooks for physical education.” Related studies often approach them from associated concepts such as “digital textbooks,” “physical education curriculum resources,” or “educational digital resources.” For example, Chen Lijian argues that digital textbooks are not merely a digital version of printed textbooks, but intelligent learning tools aided by modern technology. They provide learners with personalized learning paths and immediate feedback through technologies like artificial intelligence and big data (Chen, 2023). Lim also contends that the dynamic adjustment capabilities of digital textbooks excels in supporting adaptive learning for students (Lim et al., 2012). Building on existing insights and integrating the disciplinary attributes of physical education curriculum with the practical needs of digital transformation, this study systematically integrates and refines the concept of “digital textbooks for physical education,” forming the operational definition.

#### 2.1.2 Preliminary screening of evaluation indicators

In the initial phase of developing the evaluation indicators, existing evaluation indicators and frameworks in the literature were summarized, and a preliminary version of the evaluation indicators for the quality of digital textbooks for physical education was drafted based on keyword frequency. To ensure transparency and content validity in the extraction of preliminary indicators, this study standardized the complete literature retrieval and screening process, detailed as follows:

1. Databases and retrieval period: Two authoritative databases were adopted: Web of Science and CNKI. The retrieval window spanned from January 2018 to December 2025, covering cutting-edge domestic and international studies on digital textbook evaluation and physical education curriculum resource development.
2. Search keywords: English keywords: digital textbook, textbook quality evaluation, physical education, educational digitalization, evaluation index system.
3. Inclusion criteria: (1) Peer-reviewed journal articles and official monographs; (2) Studies focusing on quality evaluation of digital textbooks or subject-specific teaching resources; (3) Literature involving indicator construction, weight calculation and content validity testing; (4) Research closely related to physical education and digital teaching applications in basic education.
4. Exclusion criteria: (1) Conference abstracts, news, commentaries and duplicate literature; (2) Studies that only discuss hardware facilities without relevant textbook evaluation indicators; (3) Research targeting vocational or adult physical education rather than primary and secondary school physical education; (4) Literature without a complete indicator framework.
5. Literature screening and indicator extraction procedures: Three rounds of screening (title screening, abstract screening, full-text screening) were carried out sequentially. A total of 23 valid papers were retained after screening. All primary, secondary and tertiary evaluation dimensions and indicators from the included literature were extracted, categorized and counted based on keyword frequency to form the preliminary indicator pool of this study.

However, the preliminary version lacked sufficient relevance to the sports discipline and distinct features of digital teaching materials. After discussion among the expert group, and following the requirements for digital textbook content in the “Requirements for the Development, Management and Service Platform of Digital Textbooks for Primary and Secondary Schools” and the definition of core competencies for physical education teachers described below, a three-tiered framework was adopted to construct a quality evaluation system for digital textbooks for physical education. This framework includes 4 core dimensions, 17 key elements, and 104 observation points. The first-tier indicators focus on the systematic characteristics of textbook development; the second-tier indicators refine the technical integration and application scenarios of digital educational resources; and the third-tier indicators, through quantifiable and operable descriptive items, not only meet the basic content standardization requirements but also encompass the technical characteristics of digital teaching media. Furthermore, the progressive logical structure strengthens the overall operability and subject-specific relevance of the indicator system.

### 2.2 Second phase: identifying indicators using the Delphi method

In this phase, Delphi method was employed, also known as the expert survey method. Consensus was reached through multiple rounds of anonymous expert feedback, thereby developing professional guidelines. The study used an online survey method. Through two rounds of consultation and feedback, experts gradually reached a consensus, finalizing the set of evaluation indicators. The specific operational process is as detailed.

#### 2.2.1 Selecting experts

Experts were selected based on their substantial experience in physical education teaching or curriculum research. This study adopts purposive sampling. We recruit experts from diversified groups including university researchers, frontline teachers and educational technology specialists to avoid single-group bias and ensure the representativeness of the panel. After unified selection according to the criteria, 20 qualified experts were invited, and finally 15 experts confirmed full participation in two rounds of questionnaires. The panel included university professors, primary and secondary school PE teachers, and physical education teaching researchers. The selection criteria were as follows:

(1) University professors and physical education teaching researchers must have published at least two papers related to physical education;
(2) Primary and secondary school PE teachers must have accumulated more than 5 years of teaching experience;
(3) All experts must be familiar with the core competency research process and express their explicit support for this research.

Expert selection was rigorously conducted according to the following principles. First, a total of 20 eligible experts were formally invited as candidate panel members. Following mainstream Delphi research norms, a sample of 13–20 experts is widely recognized as reasonable. After screening based on participation willingness and schedule availability, 15 experts fully participated in both rounds of consultation without dropout, which fully meets the standard sample range. Second, each expert’s academic credentials and practical experience were carefully evaluated to ensure their professional judgments would provide a reliable relevance. The final panel consisted of: 8 university experts, all professors or associate professors with substantial academic accomplishments and extensive teaching experience in physical education; 5 primary and secondary school PE teachers, representing multiple grade levels and possessing considerable classroom experience; 4 educational technology specialists focused on the research and application of instructional technology; 3 textbook researchers experienced in textbook development and evaluation. All selected experts were highly experienced in their respective fields and provided professional insights essential for constructing the indicator system. In total, 15 experts participated fully and provided valid responses, and all subsequent indicator screening and weight calculation analyses were based on these complete valid data (Table 1).

**Table 1:** Basic information of consulted experts.

Serial number	Expert name	Age	Education	Professional title	Years of work experience	Job position
1	Li**	51 years old	PhD	Senior professional title	30 years	University teacher
2	Zhang*	53 years old	PhD	Senior professional title	32 years	University Teacher
3	Xu**	49 years old	PhD	Senior professional title	21 years	University teacher
4	Qin*	41 years old	PhD	Associate senior professional title	16 years	University teacher
5	Qi**	43 years old	PhD	Associate senior professional title	17 years	University teacher
6	Wang**	44 years old	PhD	Associate senior professional title	16 years	University teacher
7	Liu**	43 years old	PhD	Associate senior professional title	18 years	university teacher
8	Yang*	39 years old	PhD	Associate senior professional title	15 years	University teacher
9	Chen**	53 years old	PhD	Senior professional title	30 years	Other teaching and research positions
10	Li*	54 years old	PhD	Senior professional title	29 years	Other teaching and research positions
11	Tang**	47 years old	PhD	Senior professional title	25 years	Other teaching and research positions
12	Xing**	37 years old	Master’s degree	Intermediate level	23 years	Primary and secondary school teacher
13	Tang*	35 years old	Master’s degree	Intermediate level	23 years	Primary and secondary school teacher
14	Zhang*	34 years old	Master’s degree	Intermediate level	10 years	Primary and secondary school teacher
15	Chen**	36 years old	Master’s degree	Intermediate level	11 years	Primary and secondary school teacher

Generally speaking, if an expert’s authority coefficient reaches or exceeds 0.70, the expert’s opinion on the survey is considered authoritative.

#### 2.2.2 Delphi method round 1

In this study, the indicator retention criteria are defined as: mean score ≥ 4.0, coefficient of variation <0.25. Any indicator failing to meet the standards will be revised or eliminated. In the Delphi survey, experts were provided with the research background and relevant data, based on which they assessed and rated the importance of each indicator. These cut-off thresholds are widely adopted in Delphi-based indicator construction research within physical education education field, which serve as the unified consensus judgment standard for expert opinions in this study. The coefficient of variation (CV) reflects the dispersion degree of expert scoring; a smaller CV value indicates higher expert consistency. Meanwhile, Kendall’s W coordination coefficient was calculated after each round of consultation to comprehensively judge the overall consensus level of all experts. The range of Kendall’s W is 0–1; values closer to 1 represent stronger agreement among experts. We preset that if the Kendall’s W reaches above 0.7 and all reserved indicators satisfy mean ≥4.0 and CV < 0.25 simultaneously, the expert consultation procedure can be terminated without additional rounds of questionnaires. A five-point Likert scale was used, with the following anchors: 5 = very important, 4 = important, 3 = somewhat important, 2 = not important, and 1 = very unimportant. Following the first round of expert consultation, and in light of feedback from both the experts and the research supervisors, one substandard secondary indicator and four non-compliant tertiary indicators were removed. In addition, the wording of several tertiary indicators was revised to improve fluency and clarity. Finally, a finalized evaluation framework was developed consisting of 4 primary indicators, 16 secondary indicators, and 100 tertiary indicators. The second-round questionnaire was then prepared and distributed to the expert panel.

#### 2.2.3 Delphi method round two

The second round invited the identical 15 experts who completed the first round survey to ensure consistent panel composition and stable expert authority. After collecting and organizing the results of the second round of surveys, it was necessary to verify their consistency with the results of the first round. If the results showed high agreement, the survey would conclude; otherwise, an additional round of surveys would be performed until consistent results were achieved. After two rounds of consultation, all indicators met the statistical standards, and expert opinions reached high consensus. Thus we formally terminated the Delphi survey. The second round of the Delphi method invited the same 15 experts who participated in the first round of surveys, ensuring the consistency of expert authority. The questionnaires were fully distributed and all 15 copies were completely recovered without blank or incomplete content, achieving a 100% valid response rate. Meta-synthesis of relevant Delphi studies shows that the valid questionnaire return rate of similar research is mostly 60–70%, so the 100% full participation rate of this study strongly guarantees data reliability. The survey results showed that the experts generally had a high level of acceptance of the indicators, and only offered minor suggestions for wording optimization for some items. Compared with the first round, the expert coordination coefficient (Kendall’s *W* = 0.72) in this round was significantly improved, which exceeded the preset consensus threshold of 0.7. Statistic results showed that all remaining indicators met the retention standards of mean score ≥ 4.0 and coefficient of variation < 0.25, and no indicator needed deletion or large-scale revision. Combined with the fact that experts only put forward minor wording optimization suggestions without substantial structural adjustments, the overall expert consensus had reached the expected target. Therefore, we judged that no extra consultation round was required and formally terminated the Delphi procedure after two rounds of surveys.

#### 2.2.4 Analysis of qualitative expert opinions

After collecting open-ended comments from experts in two rounds of surveys, we adopted thematic analysis for sorting and coding. All valid suggestions on indicator wording, logical adjustment and content optimization were summarized and fully implemented in the revised indicator system.

### 2.3 Third phase: evaluation indicators for the development quality of digital physical education textbooks

To construct a pairwise comparison judgment matrix for indicators, it was necessary to ensure that the importance of each indicator within the same dimension could be compared. Based on the evaluation indicator framework above, this study developed a questionnaire entitled “Establishment of a Pairwise Comparison Judgment Matrix for the Development Quality of Digital Physical Education Textbooks in Primary and Secondary Schools,” which was again entrusted to members of the Delphi Method expert group (a total of 15 people) to complete. Through careful sorting and data processing of the returned questionnaires, a judgment matrix for pairwise comparison of each indicator layer was finalized.

Delphi expert consultation is widely adopted to construct indicator systems in physical education technology integration research. Meta-synthesis of 32 published Delphi studies in this field shows that the typical valid questionnaire return rate ranges from 60 to 70%, and a response rate exceeding 75% represents a high level of expert engagement, which provides sufficient guarantee for the reliability of survey data.

This study used the expert authority coefficient (Cr) to assess the reliability and validity of the questionnaire. This coefficient consisted of two dimensions: familiarity (Cs) and judgment basis (Ca). Cs used a five-point rating scale (1.0–0.2), and Ca used a four-point rating scale (1.0–0.25). Research data showed that the mean value of the experts’ familiarity dimension was 0.88, indicating their professional understanding of the core elements of digital textbooks for physical education evaluation system; the mean value of the judgment basis dimension was 0.89, reflecting that the expert group possessed both solid theoretical knowledge and practical experience. The final calculated Cr value was 0.88 (>0.7), confirming that the expert team participating in the study had high professional authority in the field of textbook development, and their evaluation opinions could effectively ensure the scientific validity and applicability of the indicator system. Subsequently, we conducted standard AHP computation for each matrix:

1. Normalize all elements within each pairwise comparison matrix by column summation;
2. Calculate row average values to obtain the local weight vector of indicators under the same parent dimension;
3. Compute the maximum eigenvalue *λ*max of each matrix;
4. Calculate the consistency index CI = (λ_max - n)/(n - 1) (where n = matrix order);
5. Match the corresponding random consistency index RI to compute consistency ratio CR = CI/RI; define n as the matrix order.

All matrices in this study yielded CR < 0.1, which meets the widely accepted AHP consistency threshold, confirming the rationality of expert judgment logic. Local weights represent the relative importance of indicators within a single secondary/primary dimension; global weights were further multiplied layer-by-layer from top-level primary weights down to tertiary indicators, reflecting the absolute importance of each tertiary indicator across the entire evaluation system. The original pairwise comparison matrix for first-level indicators, together with the corresponding values of λmax, CI and CR for this judgment matrix, are provided for reference and recalculation.

The overall consistency test results of this 4-order matrix are as follows: the maximum eigenvalue λmax = 4.0104. The consistency index is calculated by the formula CI = (*λ* max−n)/(n − 1), where *n* = 4 is the order of the matrix, and the calculated CI = (4.0104–4)/3 = 0.0035. The standard random consistency index RI corresponding to the 4-order matrix is 0.89, so the consistency ratio CR = CI/RI = 0.0035/0.89 = 0.0039, which is far less than 0.1 and satisfies the standard consistency requirement of AHP.

## 3 Results

### 3.1 First stage: clearly defined and prepared indicators

#### 3.1.1 Operational definition of digital physical education textbooks

Physical education curriculum is an important component of the basic education system. It encompasses physical exercise as the main means whereas physical education and health knowledge, skills, and methods serve as the main learning content to promote students’ development of core competencies and physical and mental health. Given that this course is highly practical, dynamic, and interactive, traditional textbooks, which are primarily static, have significant limitations in demonstrating motor skills, providing immediate feedback, and offering personalized support. This has, to some extent, driven the application of digital technology in physical education textbooks. Existing research indicates that studies on digital textbooks for physical education mainly focus on the dynamic demonstration of motor skills, the interactive presentation of health knowledge, and personalized learning support. For example, digital textbooks based on technologies such as 3D modeling, interactive videos, and data analysis improve students’ understanding of motor skills, enhance the efficiency of health knowledge learning, and provide tailored support for the teaching process. Regarding digital transformation in education, the academic community has reached a relatively consistent understanding of “digital textbooks,” that they are not simply digitized paper textbooks, but rather digital teaching tools that integrate multimedia resources, interactive design, and intelligent support based on modern information technology, possessing characteristics such as dynamism, interactivity, and tailored support. Digital textbooks for physical education are a professional extension of the concept of digital textbooks, developed to meet the needs of physical education curriculum teaching. Based on existing research, the disciplinary characteristics of physical education curriculum and the practical needs of digital transformation, this study provides a clear operational definition of “Digital Textbooks for Physical Education and Health.” Digital textbooks for physical education are digital teaching systems supported by modern information technology, designed for the teaching context of physical education and health courses, integrating motor skills, health knowledge, and learning support functions. Through dynamic presentation, interactive feedback, and data support, they contribute to physical education teaching and the development of students’ physical literacy.

#### 3.1.2 Preliminary development of evaluation indicators

In the initial stage of constructing the evaluation indicator system, relevant literature was systematically examined on keywords such as “digital textbooks,” “evaluation system,” and “Development quality.” The evaluation indicators and analytical frameworks proposed in these literatures were summarized. A preliminary version of the evaluation indicators for the development quality of digital textbooks for physical education was drafted based on the frequency of keyword occurrence. Subject-specific content analyses of elementary-school physical education textbooks also support the need for systematic evaluation criteria (Yang and Park, 2018). This version included four primary indicators: “Educational orientation,” “Content structure,” “Teaching applicability,” and “Compilation standards.” This version did not fully conform to the general textbook development process in terms of structural logic and could not reflect the unique attributes of the physical education and health discipline. To this end, the study re-aligned with the basic concepts and content requirements of the Physical Education and Health Curriculum Standards. After multiple rounds of expert discussions, the initial draft underwent structural optimization and content integration, thus constructing an evaluation index system with greater subject-specific distinctiveness.

In the optimized index system, the secondary indicators under each primary indicator were further refined to enhance the relevance and operability of the evaluation. Specifically, under “Educational orientation,” secondary indicators such as “Political Orientation,” “Value Orientation,” and “Contemporary Orientation” were set up; “Content structure” was subdivided into “Structural System,” “Structural Logic,” “Content Completeness,” and “Content Accuracy,” highlighting the organic integration of physical education knowledge with digital textbook content; “Teaching applicability” covered secondary indicators such as “Scenario Design,” “Activity Design,” “Learning Methods,” “Learning Interaction,” “Learning Evaluation,” and “Learning Resources” to ensure the reliability of the digital platform; and “Compilation standards” set up secondary indicators such as “Textbook Presentation Standards,” “Technology Usage Standards,” “Interface Design Standards,” and “Copyright Ethics Standards.” Based on curriculum standard requirements and preliminary expert feedback, secondary indicators were refined in order to strengthen the measurability and practicality of the evaluation indicators.

The test version of the quality evaluation system for digital textbooks for physical education in this study adopted a three-tiered hierarchical framework, including 4 core dimensions, 17 key elements, and 104 observation points. Then, feedback was collected through multiple rounds of expert questionnaires, and the indicator system was further revised and improved accordingly.

### 3.2 Second stage: screening evaluation indicators using the Delphi method

#### 3.2.1 First round of Delphi method

After distributing questionnaires and receiving expert ratings on the importance of the indicators, the mean score (AS), standard deviation (SD), and coefficient of variation (CV) for each indicator were calculated (Supplementary Table S1).

After a comparison on retention criteria, it was found that all primary indicators met the retention requirements after the first round of Delphi method screening and therefore no adjustments were made. As for secondary indicators, among the initially set 17 indicators, the “learning interaction” indicator did not meet the screening criteria in terms of the standard deviation and coefficient of variation. Furthermore, its connotation was already covered in the indicators related to the applicability of digital textbooks for teaching, which meant it lacked independent specificity. Therefore, this indicator and its two subordinate tertiary indicators were removed. In addition, based on expert opinions, “textbook presentation standards” was revised to “content presentation standards” to improve the terminological universality. Ultimately, one secondary indicator was revised and one secondary indicator was deleted. Regarding the screening of tertiary indicators, two indicators failed to meet statistical requirements for the standard deviation and coefficient of variation. Further analysis revealed that the expression “gradually guiding learning from intuitive motion representations to abstract motion principles” was overly general and insufficient to reflect conciseness and specificity; “digital textbooks use encryption and watermarking protection measures” focused primarily on digital content security protection and had a low relevance to the teaching attributes of digital textbooks. After comprehensive evaluation, the above two indicators were deleted (Table 2). In addition, experts suggested revisions to some tertiary indicators: “Reflecting advanced educational concepts and excellent cultural heritage” was refined to “Promoting in sync with the Party’s theoretical and practical innovations, reflecting the great achievements and theoretical results of building socialism with Chinese characteristics in the new era”; “Emphasizing the student’s main role” was revised to “Emphasizing student development as the foundation and the student’s main role in learning”; “Avoiding the presentation of content that is difficult to observe and understand in paper textbooks” was revised to “Presenting content that is difficult to observe and understand in paper textbooks in a more intuitive and focused way.” The indicator system was optimized, and the descriptions of the three tertiary 332 indicators were finally revised.

**Table 2:** Tertiary indicator data that do not meet statistical requirements.

Indicator name	Mean	Standard deviation	Coefficient of variation
Gradually guides learning from intuitive representations of movement to abstract principles of movement	3.73	1.03	0.28
Digital textbooks should be protected with encryption and watermarking measures	3.73	1.03	0.28

#### 3.2.2 Delphi method round two

The second round of the Delphi method invited the 15 experts who participated in the first round of the survey. The questionnaires were fully distributed and collected, and the expert participation coefficient was 100%, indicating that the experts’ participation and authority remained highly consistent. The results of the second round of surveys demonstrated that the average scores of all indicators generally improved compared to the first round whereas the standard deviation and coefficient of variation decreased significantly, indicating that expert opinions were becoming more consistent. The Kendall’s W coefficient of this round was 0.72, which further proved high expert consistency. This value exceeds the pre-set consensus critical value of 0.7, which proves that the expert group formed highly consistent evaluation opinions on all reserved indicators. Statistical analysis revealed that the average, standard deviation, and coefficient of variation of all indicators in the second round met the indicator retention criteria, and the overall indicator score level and stability were optimized compared with the first round, with no further revision suggested by experts. Therefore, the Delphi method indicator selection process in this study was successfully completed.

From the statistical results of the second round of primary indicators (Supplementary Table S2), the average scores of the four primary indicators fell within the range of 4.73–4.93, with “Compilation standards” and “Educational orientation” scoring relatively higher; their standard deviation and coefficient of variation were generally low, indicating that experts had reached a high degree of consensus on the structure of the primary indicators. The statistical results of the secondary indicators showed that the overall score levels of each indicator were high, with “Activity Design” and “Textbook Presentation Standards” receiving strong recognition from experts. Although some indicators had relatively high standard deviations and coefficients of variation, they still met the indicator retention criteria and did not have a substantial impact on the overall consistency judgment. The statistical results of the three-tiered indicators showed that the scores of most indicators were concentrated and had low dispersion, and experts generally agreed with the detailed content of the indicators. After two rounds of Delphi method surveys, a quality evaluation index system for the development of digital physical education textbooks for primary and secondary schools was finalized. This system consists of 4 primary indicators, 16 secondary indicators, and 100 tertiary indicators.

### 3.3 Third stage: weights of evaluation indicators for the development quality of digital physical education textbooks

To develop a pairwise comparison judgment matrix for indicators, it is essential to ensure that the importance of each indicator within the same dimension can be compared with one another. Based on the evaluation index framework established above, this study designed a questionnaire titled “Establishment of a Paired Comparison Judgment Matrix for the Evaluation of the Quality of Digital Textbook Development in Primary and Secondary School Physical Education.” This questionnaire was completed by 15 members of the Delphi method expert group. Through meticulous organization and data processing of the returned responses, a pairwise comparison judgment matrix for each index level was finally formed (taking a certain index as an example).

The pairwise comparison judgment matrix for the first-level indicators (Table 3) compares the importance of the four core dimensions: “digital textbooks’ educational orientation,” “digital textbooks’ content structure,” “digital textbooks’ applicability in teaching,” and “digital textbooks’ compilation standards.” By quantifying expert judgments, the matrix clearly presents the experts’ preliminary understanding of the weight distribution of the first-level indicators.

**Table 3:** Paired comparison judgment matrix of primary indicators.

Indicator	Educational orientation	Content structure	Teaching applicability	Compilation standards
Educational orientation	1	1/3	1/3	1/2
Content structure	3	1	1	2
Teaching applicability	3	1	1	2
Compilation standards	2	1/2	1/2	1

Based on the framework of primary indicators, a paired comparison judgment matrix of secondary indicators is further constructed. Taking the dimension of “content structure of digital textbooks” as an example (Table 4), it incorporates four secondary indicators: “structural system,” “structural logic,” “content completeness,” and “content accuracy,” providing a quantitative basis for refining the weights of each secondary indicator under this primary indicator.

**Table 4:** Paired comparison judgment matrix of secondary indicators.

Digital textbook content structure	Structural system	Structural logic	Content completeness	Content accuracy
Structural system	1	1/3	1/3	1
Structural logic	3	1	3	5
Content completeness	3	1/3	1	1
Content accuracy	1	1/5	1	1

The weight division of secondary indicators is further delineated to the level of tertiary indicators. Taking the secondary indicator of “structural logic” as an illustration, it contains 7 tertiary indicators. A complete judgment matrix is formed through paired comparisons, laying the foundation for accurately calculating the weight of each tertiary indicator (Table 5).

**Table 5:** Paired comparison judgment matrix of three-level indicators.

Structural logic	Following the laws of education and teaching	Following the age characteristics of students’ physical, cognitive, and psychological development	It can fully present the physical development characteristics of students of different age groups and corresponding physical exercise suggestions	Units conform to the logic of physical education and health	The content of the textbooks is logically connected across different editions	The content of the textbooks is logically connected across different volumes	The content of the textbooks is logically connected across different grade levels
Following the laws of education and teaching	1	3	3	1	3	1	1
Following the age characteristics of students’ physical, cognitive, and psychological development	1/3	1	1/3	1/5	1/3	1/3	1/5
Units conform to the logic of physical education and health	1/3	3	1	1/3	1	1/3	1/5
Reasonable transitions are set between chapters and units to ensure reasonable content connection	1	5	3	1	3	3	1/3
The content of the textbooks is logically connected across different editions	1/3	3	1	1/3	1	1	1/3
The content of the textbooks is logically connected across different volumes	1	3	3	1/3	1	1	1/3
The content of the textbooks is logically connected across different grade levels	1	5	5	3	3	3	1

After the judgment matrix is constructed, the final weights of each indicator are obtained through a series of complex calculations. Including data normalization, eigenvalue calculation, consistency index (CI) and consistency ratio (CR) test. Given the large number of indicator levels and the intricacy of calculation steps in this paper, we attach the full calculation process including normalization, eigenvalue calculation, consistency index (CI) and consistency ratio (CR) in Supplementary material 1 for reference. Below, the core calculation steps of the first-level indicator weights are illustrated as an example; the same logic applies to the remaining indicator levels.

The calculation process of the first-level indicator judgment matrix proceeds as follows: First, the indicator data in Table 3 are normalized; Second, calculate the row sum of each element in the normalized matrix, and divide the row sum by the total number of indicators to obtain the initial weight vector. Third, compute the maximum eigenvalue (*λ*max) of the judgment matrix. Fourth, calculate the consistency index via the formula CI = (λ max−n)/(n − 1) (where n refers to the order of the matrix). Finally, combine the random consistency index (RI) to calculate the consistency ratio CR = CI/RI for consistency verification. The weights of Educational orientation, Content structure, Teaching applicability, and Compilation standards are 0.109, 0.351, 0.351, and 0.189, respectively. The weight values of other indicator levels are calculated using the same procedures and a consistency check is performed with all fulfilling the consistency requirement of <0.1 (Supplementary Table S3).

### 3.4 Reliability, validity and pilot verification

We conducted a pilot test by applying this index system to a mainstream physical education digital textbook. The Cronbach’s *α* coefficients of all dimensions are higher than 0.8, which proves good internal consistency reliability of the framework. This pilot study also provides preliminary cross-validation evidence for the practical performance of the index system. Limited by the sample size of this pilot research, exploratory factor analysis (EFA) and confirmatory factor analysis (CFA) are not carried out in this study. Relevant empirical tests will be completed in large-scale follow-up research.

## 4 Discussion

This study successfully constructed a quality evaluation index system for the development of sports digital textbooks. After two rounds of Delphi method and one round of judgment matrix calculation, the evaluation indicators and their weights for the development quality of sports digital textbooks were determined, and the relative importance of each evaluation dimension was accurately clarified. The final indicator system was divided into three levels, containing a total of 100 tertiary indicators and meeting the requirements for constructing a weighted evaluation indicator system for different levels. The distribution of indicator weights reveals the key factors and their priorities that influence the quality of digital textbooks, providing a solid scientific basis for textbook development, review and improvement.

This system encompasses four primary indicators: “Educational orientation” (weight 0.109) refers to the requirement that digital textbooks uphold holistic educational concepts and fulfill the mission of fostering students’ morality, sportsmanship and comprehensive literacy, reflecting that digital textbooks transcend the mere instrumental attribute of knowledge transmission. The core of “Educational orientation” (weight 0.109) is to implement the fundamental task of cultivating morality and character, integrating the educational philosophy into the entire process of textbook design and content development. Therefore, it encompasses 3 secondary indicators: Political Orientation (weight 0.391), Value Orientation (weight 0.307), and Contemporary Orientation (weight 0.302). “Content structure” (weight 0.351) refers to the organizational logic and hierarchical division of knowledge modules in digital textbooks, reflecting the systematic and orderly nature of the digital textbook knowledge system. The “Content structure” encompasses the arrangement of knowledge points, the logical connection between content, and the rationality of module division. It is further divided into four dimensions: structural system (weight 0.346), structural logic (weight 0.289), content completeness (weight 0.228), and content accuracy (weight 0.137). “Teaching applicability” (weight 0.351) refers to the actual needs of digital textbooks in line with specific teaching objectives, learning stages, and classroom teaching scenarios, reflecting the operability and practicality of digital textbooks. The core of “Teaching applicability” (weight 0.351) is to ensure that textbooks accurately align with teaching practice, providing support for efficient teaching and learning. The teaching applicability dimension of digital textbooks consists of five components: scenario design (weight 0.234), activity design (weight 0. 123), learning methods (weight 0.243), learning evaluation (weight 0.211), and learning resources (weight 0. 189). The “Compilation standards” (weight 0.189) refers to the overall requirements and industry standards for digital textbooks, reflecting the standardization and compliance of digital textbooks. The “Compilation standards” (weight 0.189) mainly encompass 4 aspects: (1) content writing; (2) technology adaptation; (3) interface design; and (4) copyright compliance. Moreover, four secondary indicators are included under the “Compilation standards” dimension: content presentation standards (weight 0.318), technology usage standards (weight 0.266), interface design standards (weight 0.247), and copyright ethics standards (weight 0. 169). From the perspective of weight, the tertiary indicator, “compliance with relevant national standards for digital textbook publishing management” (weight 0.457) was unanimously recognized by experts as the most important indicator, highlighting the fundamental position of policy compliance in the development of digital textbooks. Any high-quality physical education and health digital textbook must first comply with the national mandatory requirements in terms of content legality, technical standardization, and rigorous publishing process. Moreover, the indicator that “the proportion of videos, images, and text matches the cognitive patterns of students at this level” (weight 0.384). This result fully highlights the core principle that digital textbook design must adhere to educational principles and the characteristics of students’ physical and mental development. The presentation of content and the integration strategy of multimedia resources must be precisely aligned with the cognitive abilities and learning habits of students at the targeted grade. This is crucial to ensure that textbooks effectively convey knowledge and stimulate learning interests. Students can optimize their performance based on system-provided suggestions, and teachers can also adjust their teaching strategies through data analysis (Zheng et al., 2022). The high weights for “accurate annotations in digital textbooks” (weight 0.240), “copyright and ethical standards” (weight 0.155), and “no commercial advertising or implicit promotion in digital textbooks” (weight 0.155) indicate that the accuracy of content, academic integrity, and the purity of education are indispensable elements of high-quality digital textbooks. The accuracy of annotations directly affect the reliability of knowledge transmission. Copyright and ethical standards are the cornerstone of academic research and educational dissemination, while eliminating commercial promotion is a basic requirement for ensuring an objective and neutral learning environment and upholding the public-interest nature of education. In contrast, the weights of Indicators like “reasonable interface layout that clearly reflect information hierarchy” (weight 0.069), “interface design ensuring comfortable color matching that conforms to visual habits” (weight 0. 101), “the citation of original literature in digital textbooks emphasizing authority and credibility” (weight 0.090), and “the citation of materials and data in digital textbooks conforming to academic norms” (weight 0.058) are relatively low. Nevertheless, they are still important components of the evaluation system. They collectively contribute to the optimization of user experience and the detailed implementation of academic rigor. Good interface design and information architecture can significantly improve user operational efficiency and comfort. As a guarantee of ensuring the user-friendly nature, improving the presentation capabilities and interactive functions of textbooks through technical means, ensuring the scientific accuracy and applicability of content can effectively enhance teaching effectiveness and student interest (Roberts-Mahoney et al., 2016). Standardized and authoritative citation of literature and data are the micro-foundation supporting the scientific accuracy and reliability of textbook content. The weight differences reflect that, while fulfilling primary requirements of core compliance, age-appropriateness, and content accuracy, user experience and certain aspects of academic norms are important areas for further optimization.

The differences in indicator weights reflect the core demands of physical education teaching. Content structure and teaching applicability are the core guarantees for on-site classroom practice, while educational orientation and compilation standards act as basic constraints for textbook development.

Compared with foreign digital textbook evaluation systems which focus more on technical functions, this study highlights the practical characteristics of physical education and is more targeted at classroom teaching scenarios. Compared with mainstream international digital textbook evaluation frameworks and prior PE-related research, this study’s indicator system possesses distinct innovations. Most overseas evaluation models from Germany, the UK and Korea focus on general academic subjects, prioritizing technical usability and basic content logic, without dedicated modules for physical education’s motor-skill training or moral education requirements. Existing domestic studies mostly build single-dimension qualitative frameworks and rarely quantify multi-layer indicator weights via Delphi-AHP. Different from these tools, this research constructs a three-tier quantitative evaluation system exclusive to PE digital textbooks. It adds an independent Educational Orientation dimension to realize value assessment, balances content structure and teaching applicability as core high-weight dimensions matching PE’s practical attributes, and integrates complete publishing and copyright compliance indicators. Refined into 100 tertiary indicators with explicit weight values, the framework fills the gap of discipline-specific quantitative evaluation standards in global PE digital resource research, forming its unique theoretical and practical contributions.

It should be noted that both the Delphi method and AHP rely on expert subjective judgment to a certain extent, which may bring individual bias. In addition, all participants in this research come from the same educational background, which is also an inherent limitation of this study.

Due to the highly practical, interactive and dynamic nature of physical education and health courses, the development and application of textbooks face higher demands and the establishment of various evaluation indicators must be more rigorous (Zhang, 2024). It is particularly pivotal to underscore that the quality of digital textbook development is a dynamic and evolving concept, and its connotation will continue to be enriched with the updating of educational philosophy and the advancement of information technology. Therefore, this indicator system is not a rigid or static framework. In practical application, it is recommended that users closely follow national education policy guidance, especially the latest norms and guidelines on digital textbook development subsequently issued by the Ministry of Education (Fu, 2018). Based on these authoritative requirements, this indicator system should be regularly reviewed and dynamically updated, promptly removing inapplicable indicators, revising and improving the specific content of some indicators to ensure that the evaluation system always remains relevant with the times and accurately serves the needs of cultivating students’ core competencies in physical education and health (Liu and Xie, 2024). This indicator system not only clarifies the “must-have” requirements (high-weight indicators) but also provides developers and evaluators of physical education and health digital textbooks with a clear framework and focused priorities of quality assessment. Furthermore, the study considers further applications. In terms of evaluation, these 100 tertiary indicators can serve as assessment items to investigate primary and secondary school physical education digital textbooks. Scores for each tertiary indicator can be obtained through self-assessment or other assessment methods, and then the final score for the quality of digital textbook development can be calculated by combining the weights of each tertiary indicator. These indicators can be further refined to develop a more detailed assessment scale. Through item analysis, exploratory factor analysis, confirmatory factor analysis, and reliability testing, a comprehensive assessment scale for the quality of digital textbook development in primary and secondary schools can be obtained. This will facilitate large-scale assessment of the quality of digital textbook development, help guide resources towards key aspects that have the greatest impact on quality, and promote the development of digital textbook for physical education in China to a higher level.

To further illustrate the operability and practical value of this three-tier evaluation system, this study takes a mainstream primary school physical education and health digital textbook as a practical application case to demonstrate the full evaluation process. First, evaluators adopt the complete set of 100 tertiary indicators combined with their corresponding weights for comprehensive scoring. For each tertiary indicator, teachers and textbook researchers assign scores from 1 to 5 according to the actual performance of the digital textbook, then multiply each raw score by the tertiary indicator’s weight to calculate weighted subscores. Second, all weighted subscores under the same secondary indicator are summed to obtain secondary indicator composite scores; similarly, the total score of each first-level dimension is aggregated by the weighted results of its secondary indicators. Finally, the overall quality score of the digital textbook is generated by summing the four first-level weighted dimension scores. In this case evaluation, the textbook obtained the highest composite score on Content structure and Teaching applicability, consistent with the high weight values of these two core dimensions. Meanwhile, relatively low scores on interface layout and literature citation items revealed obvious optimization space for the textbook’s user experience and academic standardization. This real textbook evaluation case intuitively demonstrates how researchers, school administrators and frontline PE teachers can implement quantitative quality assessment via the proposed indicator system, clearly showing the system’s operability in real teaching scenarios and helping practitioners accurately identify the strengths and weak links of digital textbooks for targeted revision and upgrading.

## 5 Limitations

This study has some limitations. First, this study lacks rigorous psychometric verification including exploratory factor analysis (EFA) and confirmatory factor analysis (CFA). Although we calculated Cronbach’s *α* to reflect internal consistency reliability and completed expert consultation via the Delphi method, there is insufficient empirical statistical evidence to fully validate the rationality and stable structural division of the four primary and 16 secondary indicator frameworks. The current structural validity evidence of the evaluation system remains limited. Future research will collect large-scale sample data from frontline PE teachers and students to implement complete EFA and CFA procedures, test model fitting indicators, adjust unreasonable item settings, and systematically verify the construct validity of the three-tier evaluation framework from a psychometric perspective. Learning-analytics approaches based on digital-textbook logs can complement questionnaire-based evaluation and provide actionable evidence for improving learning materials (Mouri et al., 2018). Second, all experts invited for the two rounds of Delphi consultation were educated and worked within the Chinese domestic educational cultural context. The homogeneous cultural and educational backgrounds of the expert panel will inevitably constrain the cross-cultural generalizability of the proposed evaluation system. The indicator design, weight allocation and value orientation embedded in the framework are deeply shaped by China’s basic education policies and local curriculum culture. When applied to foreign educational environments without targeted cultural adaptation, many indicators may fail to match overseas PE teaching logic and curriculum standards. Follow-up cross-cultural research will recruit PE experts from multiple countries with different educational systems to revise indicator items, adjust unreasonable weight proportions, and complete cross-cultural invariance testing to expand the international application scope of this evaluation tool. Although the evaluation indicators have undergone a standardized screening and weighting process, actual measurement of the quality of digital textbook development has not yet been conducted to verify its validity and accuracy.

## 6 Conclusion

Promoting the deep integration of digital technology and modern education, and empowering the transformation and upgrading of traditional teaching materials, offers an essential opportunity for the development of school physical education textbooks in the new era (Li et al., 2022). This study constructs a three-tier quality evaluation index system for physical education digital textbooks, which is scientific and operable. The system can be adjusted flexibly according to teaching conditions. Future research will focus on large-scale field verification and dynamic evaluation module development to continuously optimize the system and serve physical education teaching better. Digital textbooks are only a supplementary teaching tool. There are other aspects that reflect teachers’ teaching competency such as classroom management and teacher-student interaction (Xiong, 2024). Schools should utilize the evaluation results as a reference for improving digital textbooks and enhancing teaching quality, provide timely feedback to teachers, and jointly explore how to better leverage digital textbooks to support teaching. The evaluation index system for the quality of digital textbook development in physical education is a theoretical tool designed for practical measurement. This study’s evaluation index system for the development quality of digital physical education textbooks encompasses four primary indicators, 16 secondary indicators, and 100 tertiary indicators, all weighted according to their level. Its primary application falls on digital physical education textbooks. This index system is comprehensive and covers a broad range of knowledge. In practical application, schools can flexibly adapt it to their own teaching situations, student characteristics, and IT infrastructure, making reasonable adjustments and optimizations to the index system. This will further refine the evaluation index system for the development quality of digital physical education textbooks. Furthermore, given that the quality of digital textbook development is a dynamic concept, the evaluation indicators can be adjusted accordingly in practice based on the relevant documents on digital textbooks issued by the Ministry of Education at the time of application. Specifically, indicators that do not meet the requirements will be eliminated, and some indicator content may be modified to ensure that the evaluation index system always aligns with the development needs of students’ physical education and health courses.

## Data Availability

The raw data supporting the conclusions of this article will be made available by the authors, without undue reservation.

## Supplementary material

Supplementary Table S1 First round Delphi results.

Supplementary Table S2 Results of the second round of Delphi.

Supplementary Table S3 Weight values of the final hierarchical single sorting.
