# Supplementary material for: Developing the quality evaluation index system of digital textbooks for physical education and health
Source: Front Psychol. 2026 Sep 7;17:1825248. doi: 10.3389/fpsyg.2026.1825248 (PMC13590567; doi:10.3389/fpsyg.2026.1825248)
Supplement: Supplementary file 1 [file Table_1.docx]

**Supplementary** **Table 1. First round Delphi results.**

| **Grade** | **Indicator name** | **Standard Mean**  **deviation** | **Coefficient of variation** |
| --- | --- | --- | --- |

Primary

Digital teaching materials ’ educational

4.73

0.46

0.10

| **Grade** | **Indicator name** | **Mean** | **Standard**  **deviation** | **Coefficient of variation** |
| --- | --- | --- | --- | --- |
| indicator | orientation |  |  |  |
|  | Digital Textbook Content Structure | 4.53 | 0.83 | 0.18 |
|  | Digital Textbook Teaching Applicability | 4.80 | 0.41 | 0.09 |
|  | Digital Textbook Compilation Standards | 4.93 | 0.26 | 0.05 |
|  | Political Orientation | 4.53 | 0.83 | 0.18 |
|  | Value Orientation | 4.33 | 0.72 | 0.17 |
|  | Contemporary Orientation | 4.87 | 0.35 | 0.07 |
|  | Structural System | 4.87 | 0.35 | 0.07 |
|  | Structural Logic | 4.33 | 0.72 | 0.17 |
|  | Content Completeness | 4.20 | 0.78 | 0.19 |
|  | Content Accuracy | 4.27 | 0.80 | 0.19 |
| Secondary  Indicators | Context Design | 4.93 | 0.26 | 0.05 |
|  | Activity Design | 4.40 | 0.91 | 0.21 |
|  | Learning Methods | 4.2 | 0.94 | 0.22 |
|  | Learning Interaction | 3.67 | 1.05* | 0.29* |
|  | Learning Evaluation | 4.80 | 0.41 | 0.09 |
|  | Learning Resources | 4.47 | 0.64 | 0.14 |
|  | Textbook Presentation Standards | 4.47 | 0.74 | 0.17 |
|  | Technology Usage Standards | 3.87 | 0.92 | 0.24 |
|  | Interface Design Standards | 4.40 | 0.83 | 0.19 |

| **Grade** | **Indicator name** | **Mean** | **Standard**  **deviation** | **Coefficient of variation** |
| --- | --- | --- | --- | --- |
| Tertiary  Indicators | Copyright and Ethical Standards | 4.47 | 0.74 | 0.17 |
|  | Reflecting the Requirements of the Sinicization and Modernization of Marxism | 4.33 | 0.62 | 0.14 |
|  | Guided by Thought on Socialism with Chinese Characteristics for a New Era | 4.27 | 0.60 | 0.14 |
|  | Implement the Party's education policy and fulfill the fundamental task of  fostering virtue through education | 4.00 | 0.85 | 0.21 |
|  | Serves to cultivate socialist builders and successors who are well-rounded in moral, intellectual, physical, aesthetic, and labor education. | 4.40 | 0.51 | 0.12 |
|  | Promote and inherit advanced socialist culture and excellent traditional Chinese culture | 4.40 | 0.63 | 0.14 |
|  | Enhance identification with the great motherland, the Chinese nation, the Communist Party of China, and  socialism with Chinese characteristics | 4.47 | 0.52 | 0.12 |
|  | Reflect the basic values of the nation and the people | 4.47 | 0.52 | 0.12 |
|  | Cultivate and practice the core socialist values | 4.53 | 0.64 | 0.14 |
|  | Highlight the curriculum goals of  cultivating moral character and inspiring wisdom | 3.87 | 0.75 | 0.19 |
|  | Transmit positive energy and promote the main theme | 3.80 | 0.73 | 0.19 |
|  | Guide students to establish a correct | 4.00 | 0.93 | 0.23 |

| **Indicator name**  **Grade** | **Mean** | **Standard**  **deviation** | **Coefficient of variation** |
| --- | --- | --- | --- |
| worldview, outlook on life, and values |  |  |  |
| High-quality and tasteful, rejecting low- level tastes and vulgar orientations | 4.07 | 0.88 | 0.22 |
| Summarize the valuable experience of my country's education reform practice | 4.27 | 0.62 | 0.14 |
| Creatively transform and innovatively develop excellent traditional culture | 4.07 | 0.88 | 0.19 |
| Absorb and learn from internationally |  |  |  |
| influential and effective educational ideas and concepts | 4.47 | 0.64 | 0.14 |
| Reflecting the great achievements and |  |  |  |
| theoretical results of building socialism with Chinese characteristics in the new era | 4.33 | 0.72 | 0.17 |
| Adhering to the integrated design of the |  |  |  |
| digital textbook system for physical  education and health courses in primary, secondary, and tertiary schools | 4.27 | 0.80 | 0.19 |
| Emphasizing the students ’ central role | 4.20 | 0.87 | 0.19 |
| Paying attention to students’ diverse learning needs | 4.40 | 0.51 | 0.12 |
| Implementing the relevant requirements of the curriculum standards regarding |  |  |  |
| core competencies, curriculum | 3.93 | 0.80 | 0.20 |
| objectives, curriculum content, academic quality, and implementation suggestions |  |  |  |
| Reflecting the characteristics of physical |  |  |  |
| education and interdisciplinary learning requirements | 4.33 | 0.82 | 0.19 |

| **Grade Indicator name** | **Mean** | **Standard**  **deviation** | **Coefficient of variation** |
| --- | --- | --- | --- |
| The table of contents clearly and  intuitively displays the content structure and arrangement logic | 4.33 | 0.90 | 0.21 |
| Unit introductions concisely highlight the main theme | 4.40 | 0.63 | 0.14 |
| Each unit has clear objectives and a prominent theme | 4.67 | 0.49 | 0.10 |
| The functions of the columns are clear and the number is appropriate | 4.27 | 0.79 | 0.19 |
| Following the laws of education and teaching | 4.20 | 0.78 | 0.19 |
| Following the age characteristics of students ’ physical, cognitive, and psychological development | 4.60 | 0.74 | 0.16 |
| Units conform to the logic of physical education and health | 4.67 | 0.49 | 0.10 |
| Reasonable transitions are set between chapters and units to ensure reasonable content connection | 4.67 | 0.62 | 0.13 |
| Gradually guides learning from intuitive representations of movement to abstract principles of movement | 3.73 | 1.03* | 0.28* |
| The content of the textbooks is logically connected across different editions | 4.42 | 0.86 | 0.17 |
| The content of the textbooks is logically connected across different volumes | 4.47 | 0.83 | 0.18 |
| The content of the textbooks is logically connected across different grade levels | 4.20 | 0.78 | 0.19 |

| **Grade Indicator name** | **Mean** | **Standard**  **deviation** | **Coefficient of variation** |
| --- | --- | --- | --- |
| The content coverage is consistent with the curriculum standards | 4.0 | 0.85 | 0.21 |
| The content covers three aspects: health education, sportsmanship, and sports  culture | 4.53 | 0.74 | 0.16 |
| The content covers the main contents such as basic motor skills, physical fitness, specialized sports skills, and interdisciplinary thematic learning | 4.67 | 0.72 | 0.15 |
| The content as a whole can provide  guidance for teachers and students in learning physical education and health courses | 3.93 | 0.96 | 0.24 |
| The content reflects the forefront of  development in the discipline of physical education and health | 3.60 | 0.74 | 0.21 |
| The content can enhance flexibility and adaptability | 3.87 | 0.74 | 0.19 |
| The difficulty level of the content meets the requirements of the curriculum  standards | 4.27 | 0.80 | 0.19 |
| The content volume is appropriate | 3.87 | 0.74 | 0.21 |
| Emphasize the basic content and basic concepts of physical education,  highlighting the core content | 4.20 | 0.78 | 0.19 |
| Avoid presenting content that is difficult to observe and understand in printed  textbooks | 4.0 | 0.93 | 0.23 |
| Effectively guide students to improve their physical fitness, sports cognition, and sports skills | 3.93 | 0.80 | 0.20 |

| **Grade Indicator name** | **Mean** | **Standard**  **deviation** | **Coefficient of variation** |
| --- | --- | --- | --- |
| Effectively guide students to develop the habit of persisting in physical exercise and healthy behaviors | 4.33 | 0.72 | 0.17 |
| Effectively help students form good  sportsmanship, sports ethics, and sports character | 4.63 | 0.86 | 0.14 |
| Create authentic, effective, and typical sports and health learning situations | 3.80 | 0.73 | 0.19 |
| Focus on creating learning situations using structured knowledge and skills | 3.93 | 0.88 | 0.22 |
| Set up interesting, diverse, and life- related sports and health learning situations | 3.93 | 0.88 | 0.22 |
| Highlight the creation of practical sports and life situations | 4.07 | 0.96 | 0.24 |
| Utilize digital technology to create immersive sports and health learning situations | 4.00 | 0.76 | 0.19 |
| Guide students to solve complex problems in real situations | 3.93 | 0.70 | 0.18 |
| Stimulate students ’ interest and thirst for knowledge in sports and health learning through situations | 4.53 | 0.64 | 0.14 |
| Focus on cultivating students ’ ability to ask and solve problems | 4.73 | 0.46 | 0.10 |
| Provide students with space for  independent exploration through digital technology | 4.47 | 0.64 | 0.14 |
| Design interdisciplinary thematic and project-based learning activities | 4.67 | 0.62 | 0.13 |

| **Grade Indicator name** | **Mean** | **Standard**  **deviation** | **Coefficient of variation** |
| --- | --- | --- | --- |
| Design the number of activities  reasonably around the key points of physical education and health teaching | 4.33 | 0.98 | 0.23 |
| Emphasize the design ofphysical  education and health activities that combine in-school and out-of-school learning | 4.07 | 0.89 | 0.29 |
| Emphasize the design ofphysical education and health activities that combine in-class and out-of-class learning | 3.87 | 0.74 | 0.19 |
| Emphasize the design ofphysical  education and health activities that combine’ online and offline learning | 3.87 | 0.92 | 0.24 |
| Emphasize the transformation of studentslearning methods | 4.33 | 0.82 | 0.19 |
| Emphasize the transformation of teachers ’ teaching methods | 4.60 | 0.74 | 0.16 |
| Guide students to conduct self-directed learning | 3.93 | 1.16 | 0.30 |
| Guide students to conduct collaborative learning | 4.47 | 0.83 | 0.18 |
| Guide students to conduct inquiry-based learning | 4.80 | 0.41 | 0.08 |
| Fully leverage the role of information technology in the transformation of learning methods | 4.13 | 0.84 | 0.20 |
| Support personalized learning and differentiated teaching | 4.60 | 0.74 | 0.16 |
| Enable students to interact with the | 4.00 | 1.00* | 0.25* |

| **Grade Indicator name** | **Mean** | **Standard**  **deviation** | **Coefficient of variation** |
| --- | --- | --- | --- |
| textbook |  |  |  |
| Enable teachers to interact with the textbook | 4.00 | 1.00* | 0.25* |
| Based on academic quality | 4.67 | 0.49 | 0.10 |
| Emphasis on cultivating and developing core competencies in physical education | 4.60 | 0.74 | 0.16 |
| Emphasis on diversifying evaluation methods | 4.20 | 0.78 | 0.19 |
| Focus on students ’ level progression at each stage  Emphasis on diversifying evaluation | 4.27 | 0.79 | 0.19 |
| subjects such as teacher evaluation, student self-evaluation, and peer  evaluation | 4.07 | 0.89 | 0.29 |
| Emphasis on feedback and improvement of evaluation results | 3.87 | 0.74 | 0.19 |
| Provide ample learning resources for teachers and students | 4.00 | 0.93 | 0.23 |
| Guide teachers to effectively utilize and proactively develop teaching resources | 4.40 | 0.63 | 0.14 |
| Advocate for resource sharing | 3.87 | 0.92 | 0.24 |
| Avoid the appearance of commercial |  |  |  |
| teaching aids such as website addresses and QR codes | 4.33 | 0.82 | 0.19 |
| Rational use of digital resources | 4.60 | 0.74 | 0.16 |
| All symbols, logos, fonts, and sizes conform to national standards | 3.93 | 1.16 | 0.30 |

| **Grade Indicator name** | **Mean** | **Standard**  **deviation** | **Coefficient of variation** |
| --- | --- | --- | --- |
| Accurately draw the Party flag, Party  emblem, national flag, national emblem, and politically significant buildings | 4.27 | 0.80 | 0.19 |
| Language expression methods meet  professional and aesthetic requirements | 4.73 | 0.46 | 0.10 |
| Present the new development of  language in the digital age in conjunction with the characteristics of sports  discipline | 4.07 | 0.80 | 0.19 |
| The cover highlights the characteristics of sports and health discipline | 4.20 | 0.76 | 0.18 |
| The images, sound, and subtitles are  organically connected, coordinated, and played smoothly | 4.33 | 0.72 | 0.17 |
| The use of technology complies with relevant national laws and regulations | 4.13 | 0.74 | 0.18 |
| Resources can be shared, interoperable, and transferred across platforms between computers, networks, operating systems, and applications | 4.73 | 0.60 | 0.13 |
| Standardize the management of data collection, use, and storage to protect user data privacy | 4.60 | 0.63 | 0.14 |
| Provide sufficient technical methods,  means, and support such as virtual  simulation, 3D, and AI to help teachers and students obtain information on  physical education and health curriculum | 4.67 | 0.62 | 0.13 |
| Ensure that the interactive design is  simple, easy to use, and conforms to the cognitive habits of teachers and students | 4.60 | 0.63 | 0.14 |

| **Grade Indicator name** | **Mean** | **Standard**  **deviation** | **Coefficient of variation** |
| --- | --- | --- | --- |
| Provide sufficient text, audio, and video materials to assist teaching | 4.67 | 0.49 | 0.10 |
| The interface design is aesthetically  pleasing and reflects the characteristics of the Physical Education and Health discipline. | 4.40 | 0.63 | 0.14 |
| The interface design embodies modern aesthetics while inheriting and promoting the spirit of traditional Chinese  aesthetics. | 4.33 | 0.90 | 0.21 |
| The interface design possesses  educational value and positive aesthetic requirements. | 4.33 | 0.82 | 0.19 |
| The interface layout is reasonable and clearly reflects the information  hierarchy. | 4.60 | 0.51 | 0.11 |
| The proportion of videos, images, and  text conforms to the cognitive patterns of students at this level. | 4.20 | 0.87 | 0.19 |
| The interface design ensures comfortable color matching that conforms to visual habits. | 4.27 | 0.80 | 0.19 |
| The citation of original literature in  digital textbooks emphasizes authority and credibility. | 4.33 | 0.72 | 0.17 |
| Digital textbooks use encryption and watermarking protection measures. | 3.73 | 1.03* | 0.28* |
| The cited materials and data in digital textbooks comply with academic norms. | 4.07 | 0.88 | 0.19 |
| The footnotes in digital textbooks are accurate and error-free. | 4.00 | 0.77 | 0.19 |

| **Grade** | **Indicator name** | **Mean** | **Standard**  **deviation** | **Coefficient of variation** |
| --- | --- | --- | --- | --- |

| Digital textbooks contain no commercial advertisements or implicit promotions. | 4.07 | 0.88 | 0.22 |
| --- | --- | --- | --- |
| They comply with relevant national  standards for the management of digital textbook publishing. | 3.80 | 0.73 | 0.19 |

**Supplementary** **Table 2. Results of the second round of Delphi.**

| **Grade** | **Indicator name** | **Coefficient**  **Standard**  **deviation**  **Mean**  **of variation** | | |
| --- | --- | --- | --- | --- |
| Primary  indicator | Digital teaching materials ’ educational orientation | 4.87 | 0.35 | 0.05 |
|  | Digital Textbook Content Structure | 4.80 | 0.41 | 0.13 |
|  | Digital Textbook Teaching Applicability | 4.73 | 0.60 | 0.13 |
|  | Digital Textbook Compilation Standards | 4.93 | 0.26 | 0.05 |
| Secondary  Indicators | Political Orientation | 4.67 | 0.49 | 0.11 |
|  | Value Orientation | 4.47 | 0.64 | 0.14 |
|  | Contemporary Orientation | 4.87 | 0.35 | 0.07 |

| **Grade** | **Indicator name** | **Mean** | **Standard**  **deviation** | **Coefficient**  **of variation** |
| --- | --- | --- | --- | --- |
| Tertiary  Indicators | Structural System | 4.87 | 0.35 | 0.07 |
|  | Structural Logic | 4.80 | 0.41 | 0.09 |
|  | Content Completeness | 4.73 | 0.46 | 0.10 |
|  | Content Accuracy | 4.40 | 0.63 | 0.14 |
|  | Context Design | 4.53 | 0.64 | 0.14 |
|  | Activity Design | 4.93 | 0.26 | 0.05 |
|  | Learning Methods | 4.87 | 0.35 | 0.07 |
|  | Learning Evaluation | 4.40 | 0.74 | 0.17 |
|  | Learning Resources | 4.60 | 0.63 | 0.14 |
|  | Textbook Presentation Standards | 4.93 | 0.26 | 0.05 |
|  | Technology Usage Standards | 4.60 | 0.63 | 0.14 |
|  | Interface Design Standards | 4.40 | 0.74 | 0.17 |
|  | Copyright and Ethical Standards | 4.40 | 0.63 | 0.14 |
|  | Reflecting the Requirements of the  Sinicization and Modernization of Marxism | 4.27 | 0.79 | 0.19 |
|  | Guided by Thought on Socialism with Chinese Characteristics for a New Era | 4.20 | 0.78 | 0.19 |
|  | Implement the Party's education policy and fulfill the fundamental task of fostering virtue through education | 4.60 | 0.74 | 0.16 |
|  | Serving to cultivate socialist builders and successors who are well-rounded in moral, intellectual, physical, aesthetic, and labor | 4.67 | 0.49 | 0.10 |

| **Indicator name**  **Grade** | **Mean** | **Standard**  **deviation** | **Coefficient**  **of variation** |
| --- | --- | --- | --- |
| education |  |  |  |
| Promote and inherit advanced socialist culture and excellent traditional Chinese culture | 4.67 | 0.62 | 0.13 |
| Enhance identification with the great |  |  |  |
| motherland, the Chinese nation, the  Communist Party of China, and socialism with Chinese characteristics | 4.80 | 0.41 | 0.08 |
| Reflect the basic values of the nation and the people | 4.60 | 0.74 | 0.16 |
| Cultivate and practice the core socialist values | 4.13 | 0.84 | 0.20 |
| Highlight the curriculum goals of cultivating moral character and inspiring wisdom | 4.60 | 0.51 | 0.11 |
| Transmit positive energy and promote the main theme | 4.40 | 0.63 | 0.14 |
| Guide students to establish a correct worldview, outlook on life, and values | 4.33 | 0.72 | 0.17 |
| High-quality and tasteful, rejecting low-level tastes and vulgar orientations | 4.20 | 0.68 | 0.16 |
| Summarize the valuable experience of my country's education reform practice | 4.20 | 0.68 | 0.16 |
| Creatively transform and innovatively develop excellent traditional culture | 4.27 | 0.59 | 0.14 |
| Absorb and learn from internationally |  |  |  |
| influential and effective educational ideas and concepts | 4.40 | 0.74 | 0.17 |
| Promoting in sync with the Party's theoretical |  |  |  |
| and practical innovations, reflecting the great achievements and theoretical results of the development of socialism with Chinese | 4.47 | 0.64 | 0.14 |

| **Grade Indicator name** | **Mean** | **Standard**  **deviation** | **Coefficient**  **of variation** |
| --- | --- | --- | --- |
| characteristics in the new era |  |  |  |
| Adhering to the integrated design of the digital |  |  |  |
| textbook system for physical education and health courses in primary, secondary, and tertiary schools | 4.33 | 0.62 | 0.14 |
| Emphasizing student development as the |  |  |  |
| foundation and the main role of student learning | 4.47 | 0.64 | 0.14 |
| Paying attention to students ’ diverse learning needs | 4.33 | 0.62 | 0.14 |
| Implementing the relevant requirements of the |  |  |  |
| curriculum standards regarding core |  |  |  |
| competencies, curriculum objectives, | 4.47 | 0.64 | 0.14 |
| curriculum content, academic quality, and implementation suggestions |  |  |  |
| Reflecting the characteristics of physical |  |  |  |
| education and interdisciplinary learning requirements | 4.33 | 0.62 | 0.14 |
| The table of contents clearly and intuitively |  |  |  |
| displays the content structure and arrangement logic | 4.40 | 0.63 | 0.14 |
| Unit introductions concisely highlight the main theme | 4.80 | 0.41 | 0.09 |
| Each unit has clear objectives and a prominent theme | 4.33 | 0.62 | 0.14 |
| The functions of the columns are clear and the number is appropriate | 4.73 | 0.59 | 0.12 |
| Following the laws of education and teaching | 4.60 | 0.63 | 0.14 |
| Following the age characteristics of students ’ physical, cognitive, and psychological | 4.40 | 0.63 | 0.14 |

| **Grade Indicator name** | **Mean** | **Standard**  **deviation** | **Coefficient**  **of variation** |
| --- | --- | --- | --- |
| development  Units conform to the logic ofphysical education and health | 4.47 | 0.64 | 0.14 |
| Reasonable transitions are set between chapters and units to ensure reasonable content connection | 4.27 | 0.59 | 0.13 |
| The content of the textbooks is logically connected across different editions | 4.20 | 0.68 | 0.16 |
| The content of the textbooks is logically connected across different volumes | 4.33 | 0.72 | 0.17 |
| The content of the textbooks is logically connected across different grade levels | 4.47 | 0.64 | 0.14 |
| The content coverage is consistent with the curriculum standards | 4.27 | 0.59 | 0.13 |
| The content covers three aspects: health  education, sportsmanship, and sports culture | 4.20 | 0.68 | 0.16 |
| The content covers the main contents such as basic motor skills, physical fitness, specialized sports skills, and interdisciplinary thematic  learning | 4.33 | 0.72 | 0.17 |
| The content as a whole can provide guidance for teachers and students in learning physical education and health curriculum | 4.47 | 0.52 | 0.12 |
| The content reflects the forefront of  development in the discipline of physical education and health | 4.40 | 0.51 | 0.12 |
| The content can enhance flexibility and adaptability | 4.60 | 0.51 | 0.11 |
| The difficulty level of the content meets the | 4.33 | 0.62 | 0.14 |

| **Grade Indicator name** | **Mean** | **Standard**  **deviation** | **Coefficient**  **of variation** |
| --- | --- | --- | --- |
| requirements of the curriculum standards |  |  |  |
| The content volume is appropriate | 4.53 | 0.64 | 0.14 |
| Emphasize the basic content and basic |  |  |  |
| concepts of physical education, highlighting the core content | 4.60 | 0.51 | 0.11 |
| Presenting content that is difficult to observe |  |  |  |
| and understand in paper textbooks more intuitively and with emphasis | 4.33 | 0.62 | 0.14 |
| Effectively guide students to improve their |  |  |  |
| physical fitness, sports cognition, and sports skills | 4.53 | 0.64 | 0.14 |
| Effectively guide students to develop the habit |  |  |  |
| of persisting in physical exercise and healthy behaviors | 4.33 | 0.72 | 0.17 |
| Effectively help students form good |  |  |  |
| sportsmanship, sports ethics, and sports character | 4.27 | 0.59 | 0.14 |
| Create authentic, effective, and typical sports and health learning situations | 4.27 | 0.86 | 0.23 |
| Focus on creating learning situations using structured knowledge and skills | 4.17 | 0.81 | 0.23 |
| Set up interesting, diverse, and life-related sports and health learning situations | 4.10 | 0.56 | 0.09 |
| Highlight the creation of practical sports and life situations | 4.53 | 0.71 | 0.19 |
| Utilize digital technology to create immersive sports and health learning situations | 4.63 | 0.52 | 0.13 |
| Guide students to solve complex problems in real situations | 4.33 | 0.62 | 0.14 |

| **Grade Indicator name** | **Mean** | **Standard**  **deviation** | **Coefficient**  **of variation** |
| --- | --- | --- | --- |
| Stimulate students ’ interest and thirst for knowledge in sports and health learning through situations | 4.73 | 0.46 | 0.10 |
| Focus on cultivating students ’ ability to ask and solve problems | 4.80 | 0.41 | 0.08 |
| Provide students with space for independent exploration through digital technology | 4.87 | 0.35 | 0.07 |
| Design interdisciplinary thematic and project- based learning activities | 4.53 | 0.64 | 0.14 |
| Design the number of activities reasonably around the key points ofphysical education and health teaching | 4.27 | 0.59 | 0.14 |
| Emphasize the design of physical education and health activities that combine in-school and out-of-school learning | 4.53 | 0.72 | 0.14 |
| Emphasize the design of physical education and health activities that combine in-class and out-of-class learning | 4.47 | 0.59 | 0.13 |
| Emphasize the design of physical education and health activities that combine online and offline learning | 4.67 | 0.68 | 0.16 |
| Emphasize the transformation of students ’ learning methods | 4.73 | 0.64 | 0.21 |
| Emphasize the transformation of teachers ’ teaching methods | 4.40 | 0.65 | 0.11 |
| Guide students to conduct self-directed learning | 4.60 | 0.45 | 0.08 |
| Guide students to conduct collaborative learning | 4.80 | 0.41 | 0.07 |

| **Grade Indicator name** | **Mean** | **Standard**  **deviation** | **Coefficient**  **of variation** |
| --- | --- | --- | --- |
| Guide students to conduct inquiry-based learning | 4.73 | 0.82 | 0.11 |
| Fully leverage the role of information  technology in the transformation of learning methods | 4.73 | 0.72 | 0.12 |
| Support personalized learning and differentiated teaching | 4.33 | 0.54 | 0.09 |
| Based on academic quality | 4.33 | 0.61 | 0.13 |
| Emphasis on cultivating and developing core competencies in physical education | 4.40 | 0.36 | 0.17 |
| Emphasis on diversifying evaluation methods | 4.13 | 0.35 | 0.16 |
| Focus on students ’ level progression at each stage | 4.47 | 0.41 | 0.12 |
| Emphasis on diversifying evaluation subjects such as teacher evaluation, student self-  evaluation, and peer evaluation | 4.47 | 0.46 | 0.11 |
| Emphasis on feedback and improvement of evaluation results | 4.33 | 0.35 | 0.13 |
| Provide ample learning resources for teachers and students | 4.93 | 0.56 | 0.09 |
| Guide teachers to effectively utilize and proactively develop teaching resources | 4.87 | 0.52 | 0.12 |
| Advocate for resource sharing | 4.80 | 0.42 | 0.15 |
| Avoid the appearance of commercial teaching aids such as website addresses and QR codes | 4.73 | 0.63 | 0.16 |
| Rational use of digital resources | 4.60 | 0.32 | 0.21 |

| **Grade Indicator name** | **Mean** | **Standard**  **deviation** | **Coefficient**  **of variation** |
| --- | --- | --- | --- |
| All symbols, logos, fonts, and sizes conform to national standards | 4.53 | 0.25 | 0.18 |
| Accurately draw the Party flag, Party emblem, national flag, national emblem, and politically significant buildings | 4.60 | 0.72 | 0.14 |
| Language expression methods meet  professional and aesthetic requirements | 4.60 | 0.63 | 0.15 |
| Present the new development of language in the digital age in conjunction with the  characteristics of sports discipline | 4.87 | 0.41 | 0.16 |
| The cover highlights the characteristics of sports and health discipline | 4.80 | 0.64 | 0.11 |
| The images, sound, and subtitles are  organically connected, coordinated, and played smoothly | 4.67 | 0.49 | 0.08 |
| The use of technology complies with relevant national laws and regulations | 4.87 | 0.46 | 0.13 |
| Resources can be shared, interoperable, and transferred across platforms between  computers, networks, operating systems, and applications | 4.73 | 0.51 | 0.11 |
| Standardize the management of data  collection, use, and storage to protect user data privacy | 4.60 | 0.26 | 0.16 |
| Provide sufficient technical methods, means, and support such as virtual simulation, 3D, and AI to help teachers and students obtain information on physical education and health curriculum | 4.80 | 0.64 | 0.14 |
| Ensure that the interactive design is simple, easy to use, and conforms to the cognitive | 4.73 | 0.62 | 0.12 |

| **Grade Indicator name** | **Mean** | **Standard**  **deviation** | **Coefficient**  **of variation** |
| --- | --- | --- | --- |
| habits of teachers and students |  |  |  |
| Provide sufficient text, audio, and video materials to assist teaching | 4.33 | 0.49 | 0.13 |
| The interface design is aesthetically pleasing |  |  |  |
| and reflects the characteristics of the Physical Education and Health discipline. | 4.40 | 0.64 | 0.16 |
| The interface design embodies modern |  |  |  |
| aesthetics while inheriting and promoting the spirit of traditional Chinese aesthetics. | 4.33 | 0.62 | 0.18 |
| The interface design possesses educational value and positive aesthetic requirements. | 4.47 | 0.64 | 0.18 |
| The interface layout is reasonable and clearly reflects the information hierarchy.  The proportion of videos, images, and text | 4.20 | 0.74 | 0.17 |
| conforms to the cognitive patterns of students at this level. | 4.56 | 0.72 | 0.11 |
| The interface design ensures comfortable color matching that conforms to visual habits.  The citation of original literature in digital | 4.17 | 0.62 | 0.09 |
| textbooks emphasizes authority and credibility. | 4.45 | 0.63 | 0.15 |
| The cited materials and data in digital textbooks comply with academic norms. | 4.69 | 0.41 | 0.13 |
| The footnotes in digital textbooks are accurate and error-free. | 4.56 | 0.49 | 0.15 |
| Digital textbooks contain no commercial advertisements or implicit promotions. | 4.32 | 0.53 | 0.09 |
| They comply with relevant national standards for the management of digital textbook | 4.60 | 0.52 | 0.17 |

| **Grade** | **Indicator name** | **Mean** | **Standard**  **deviation** | **Coefficient**  **of variation** |
| --- | --- | --- | --- | --- |
| publishing. | | | | |

**Supplementary** **Table 3. Weight Values of the Final Hierarchical Single Sorting.**

| First-level  Dimension | Second-level  Dimension | Third-level Dimension |
| --- | --- | --- |
| Digital Textbook's  Educational  Orientation (0. 109) | Political  Orientation  (0.391) | Reflecting the Requirements of the Sinicization and Modernization of Marxism (0.071)  Guided by Thought on Socialism with Chinese Characteristics for a New Era (0. 186)  Implementing the Party's Education Policy and  Fulfilling the Fundamental Task of Fostering Morality and Cultivating Talent (0.054)  Serving the Cultivation of Socialist Builders and  Successors with All-round Development in Morality, Intelligence, Physical Fitness, Aesthetics, and Labor (0.312)  Promoting and Inheriting Advanced Socialist Culture and Excellent Traditional Chinese Culture (0.137) |

| First-level  Dimension | Second-level  Dimension | Third-level Dimension |
| --- | --- | --- |
| Digital textbook  content structure  (0.351) | Value Orientation  (0.307)  Guided by the times (0.302)  Structural system  (0.346) | Enhancing Identification with the Great Motherland, the Chinese Nation, the Communist Party of China, and  Socialism with Chinese Characteristics (0.240)  Reflects the basic values of the nation and the people (0.039)  Cultivates and practices the core socialist values (0.337)  Highlights the goals of moral education, enlightenment, and wisdom (0.092)  Transmits positive energy and promotes the main theme (0.175)  Guides students to establish correct worldviews, outlooks on life, and values (0.048)  High-class taste and refined style, rejecting low-level interests and vulgar orientations (0.309)  Summarizes the valuable experience of my country's educational reform practice (0.407)  Creatively transforms and innovatively develops excellent traditional culture (0.235)  Absorbs and learns from internationally influential and effective educational ideas and concepts (0.179)  In line with the Party's theoretical and practical  innovations, reflecting the great achievements and theoretical results of building socialism with Chinese characteristics in the new era (0.179)  Adhering to the integrated design of the digital textbook system for physical education and health courses in  primary, secondary, and tertiary schools (0.158)  Emphasizing student development as the foundation and |

| First-level  Dimension | Second-level  Dimension | Third-level Dimension |
| --- | --- | --- |
|  | Structural logic  (0.289) | the student's main role in learning (0. 123)  Paying attention to students ’ diverse learning needs (0. 141)  Implementing the relevant requirements of the  curriculum standards regarding core competencies, curriculum objectives, curriculum content, academic quality, and implementation suggestions (0.137)  Reflecting the characteristics of physical education and interdisciplinary learning requirements (0. 114)  The table of contents clearly and intuitively displays the content structure and arrangement logic (0.096)  Unit introductions concisely highlight the main theme (0.085)  Each unit has clear objectives and a prominent theme (0. 109)  Column functions are clear and the number is appropriate (0.037)  Follows the laws of education and teaching (0.246)  Follows the age characteristics of students ’ physical, cognitive, and psychological development (0.273)  Units conform to the logic of the Physical Education and Health discipline (0.195)  Reasonable transitions are set between chapters and units to ensure reasonable content connection (0.031)  Reasonable connection of textbook content between different editions (0.075)  Reasonable connection of textbook content between |

| First-level  Dimension | Second-level  Dimension | Third-level Dimension |
| --- | --- | --- |
| Content complete  (0.228) | | different volumes (0.058)  Reasonable connection of textbook content between different grade levels (0.062)  Content coverage is consistent with the curriculum standards (0. 122)  Content covers three aspects: health education, sportsmanship, and sports culture (0.211)  Content covers the main contents such as basic motor skills, physical fitness, specialized sports skills, and interdisciplinary thematic learning (0.253)  Overall, the content can provide guidance for teachers and students in learning physical education and health curriculum (0.059)  Content reflects the forefront of development in physical education and health disciplines (0. 101)  Content enhances flexibility and adaptability (0.253)  Content difficulty level meets curriculum standard requirements (0.035)  Content volume is appropriate (0.267)  Focuses on basic content and concepts ofphysical education, highlighting core content (0. 123) |
| Content is  accurate (0.137) | | Presents content that is difficult to observe and  understand in printed textbooks more intuitively and with emphasis (0.076)  Effectively guides students to improve their physical fitness, sports cognition, and sports skills (0.312)  Effectively guides students to develop the habit of persisting in physical exercise and healthy behaviors |

| First-level  Dimension | Second-level  Dimension | Third-level Dimension |
| --- | --- | --- |
| Digital textbook  teaching  applicability (0.351) | Context design  (0.234)  Activity design  (0. 123) | (0.046)  Effectively helps students form good sportsmanship, sports ethics, and sports character (0. 141)  Creating realistic, effective, and typical physical education and health learning contexts (0. 113)  Emphasizing the creation of learning contexts using structured knowledge and skills (0.137)  Setting up fun, diverse, and life-related physical education and health learning contexts (0.105)  Highlighting the creation of practical sports and life contexts (0. 146)  Utilizing digital technology to create immersive physical education and health learning contexts (0. 128)  Guiding students to solve complex problems in real-life contexts (0. 169)  Stimulating students ’ interest and thirst for knowledge in physical education and health learning through contexts (0. 102)  Emphasizing the cultivation of students ’ ability to ask and solve problems (0.098)  Providing students with space for independent exploration through digital technology (0.156)  Designing interdisciplinary thematic and project-based learning activities (0. 124)  Rationally designing the number of activities around the key points of physical education and health teaching  (0.117)  Emphasizing the design ofphysical education and health |

| First-level  Dimension | Second-level  Dimension | Third-level Dimension |
| --- | --- | --- |
| Learning methods  (0.243) | | activities that combine in-school and out-of-school learning (0. 143)  Emphasizing the design ofphysical education and health activities that combine in-class and out-of-class learning (0.165)  Emphasizing the design ofphysical education and health activities that combine online and offline learning  (0.197)  Emphasizing the transformation of students’ learning methods (0.085)  Emphasizing the transformation of teachers’ teaching methods (0. 164)  Guiding students to conduct independent learning (0.137)  Guiding students to conduct cooperative learning (0. 106)  Guiding students to conduct inquiry-based learning (0. 123)  Fully leveraging the role of information technology in the transformation of learning methods (0.178)  Supporting personalized learning and differentiated teaching (0.107)  Based on academic quality (0.062)  Emphasizing the cultivation and development of core |
| Learning  evaluation (0.211) | | competencies in physical education (0.197)  Emphasizing the diversification of evaluation methods (0.048)  Focusing on the level progression of students at different |

| First-level  Dimension | Second-level  Dimension | Third-level Dimension |
| --- | --- | --- |
| Standards for digital textbook  compilation (0. 189) | Learning resources  (0. 189)  Standards for  content  presentation  (0.318) | stages of learning (0.285)  Emphasizing the diversification of evaluation subjects such as teacher evaluation, student self-evaluation, and peer evaluation (0.093)  Emphasizing feedback and improvement of evaluation results (0.315)  Providing teachers and students with sufficient learning resources (0.158)  Guide teachers to effectively utilize and proactively develop teaching resources (0.043)  Advocate resource sharing (0.413)  Avoid the appearance of commercial teaching aids such as website addresses and QR codes (0.266)  Rationally utilize digital resources (0. 120)  Various symbol identifiers, font sizes, etc. comply with the national general standard (0.195)  Accurately draw the Party flag, Party emblem, national flag, national emblem, and politically significant  buildings (0.423)  The language expression and methods meet the  requirements of professionalism and aesthetics (0.052)  Combining the characteristics of sports disciplines to present new developments in digital era language  (0.147)  The cover highlights the characteristics of sports and health disciplines (0.107)  Organic connection, coordination, and smooth playback |

| First-level  Dimension | Second-level  Dimension | Third-level Dimension |
| --- | --- | --- |
| Technical Usage  Specification  (0.266) | | of visuals, sound, subtitles, etc. (0.076)  The use of technology complies with relevant national laws and regulations (0.083)  Resources can be shared, interoperable, and transferred across platforms between computers, networks,  operating systems, and applications (0.219)  Standardize the collection, use, and storage of  management data to protect user data privacy (0.037)  Provide sufficient technical methods, means, and  support such as virtual simulation, 3D, and AI to help teachers and students obtain information on physical education and health courses (0.346)  Ensure the interactive design is simple and easy to use, conforming to the cognitive habits of teachers and  students (0.105)  Provide sufficient materials of various types, such as graphics, audio, and video, to assist teaching (0.200)  The interface design is beautiful and elegant, reflecting the characteristics of the physical education and health discipline (0.023)  The interface design can reflect modern aesthetic style on the basis of inheriting and carrying forward the |
| Interface design is  standardized  (0.247) | | traditional Chinese aesthetic spirit (0.275)  The interface design has educational value and positive aesthetic requirements (0. 148)  The interface design layout is reasonable and can clearly reflect the information hierarchy (0.069)  The matching ratio of videos, images, text, etc. conforms to the cognitive patterns of students at this level (0.384) |

| First-level  Dimension | Second-level  Dimension | Third-level Dimension |
| --- | --- | --- |
|  | Copyright Ethics Standards (0. 169) | Interface design ensures comfortable color matching that conforms to visual habits (0. 101)  The citation of original literature in digital textbooks emphasizes authority and credibility (0.090)  The cited materials and data in digital textbooks comply with academic standards (0.058)  The annotations appearing in digital textbooks are accurate and correct (0.240)  There are no commercial advertisements or implicit promotions in digital textbooks (0.155)  Comply with the relevant standards of national digital textbook publishing management (0.457) |
